# Gradenigo's Syndrome with Carotid Septic Stenosis

**DOI:** 10.1155/2020/9439184

**Published:** 2020-02-19

**Authors:** Ana Sousa Menezes, Daniela Ribeiro, Filipa Balona, Ricardo Maré, Cátia Azevedo, Jaime Rocha, Luís Dias

**Affiliations:** ^1^Department of Otorhinolaryngology-Head and Neck Surgery, Hospital de Braga, Braga, Portugal; ^2^Department of Pediatrics, Hospital de Braga, Braga, Portugal; ^3^Department of Neurology, Hospital de Braga, Braga, Portugal; ^4^Department of Neuroradiology, Hospital de Braga, Braga, Portugal

## Abstract

Gradenigo's syndrome was firstly described in 1907 by Giusseppe Gradenigo and is defined as the clinical triad of suppurative otitis media, ipsilateral abducens nerve palsy, and pain in the distribution of the first and the second branches of the trigeminal nerve. Since the advent of antibiotics, the incidence of this potentially life-threatening complication has diminished, but occasional cases still occur. We herein report a pediatric case of otitis media associated with Gradenigo's syndrome complicated by ipsilateral septic cavernous sinus thrombosis and infectious arteritis of the internal carotid artery.

## 1. Introduction

Gradenigo's syndrome (GS) has always been considered a classical condition, well known by many but currently seen by very few. In fact, GS has become a rare diagnosis in the modern antibiotic era [1–3].

Although being a rare and relatively forgotten process of years past, GS can be potentially life-threatening with significant mortality. Cerebral vascular thrombosis is a serious neurological complication of otitis media and occurs secondary to spread of the infection to the underlying bone.

To the best of our knowledge, very few case reports have been published describing venous sinus thrombosis in GS, and only two reported the commitment of the carotid artery [4–10].

We believe it is of utmost importance to raise awareness of these vascular complications of GS, which may allow for early detection and treatment of one of the most serious complications of otitis media. Additionally, this case report emphasizes the importance of appropriate radiological investigation and timely treatment, which in our case included surgery and medical therapy.

## 2. Case Presentation

**Figure 1:**
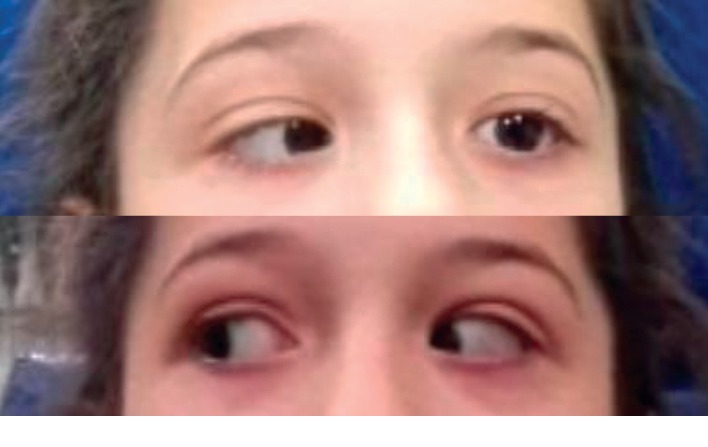
Left lateral rectus palsy as demonstrated by the patient's inability to abduct her left eye.

An 8-year-old girl was admitted to the emergency department with a 10-day history of left-sided otalgia, otorrhea, and ipsilateral hearing loss and new onset strabismus and binocular diplopia since the day of admission. She was under paracetamol and ibuprofen every 4 hours since the beginning of the disease, with fever noticed only in day 4 and had taken 1 dose of cotrimoxazole without medical prescription. She had been vomiting since the previous night, with no headache or ocular pain. Her medical history was unremarkable. Physical examination revealed suppurative left-sided acute otitis media, bilateral conductive hearing loss on acumetry with tuning forks, ipsilateral abducens nerve palsy (Figure 1), and a fever of 38.0°C. Sensation to light touch and pinprick in the distribution of the fifth (trigeminal) cranial nerve was preserved. No other focal neurological deficit was present. Meningeal signs were negative.

**Figure 2:**
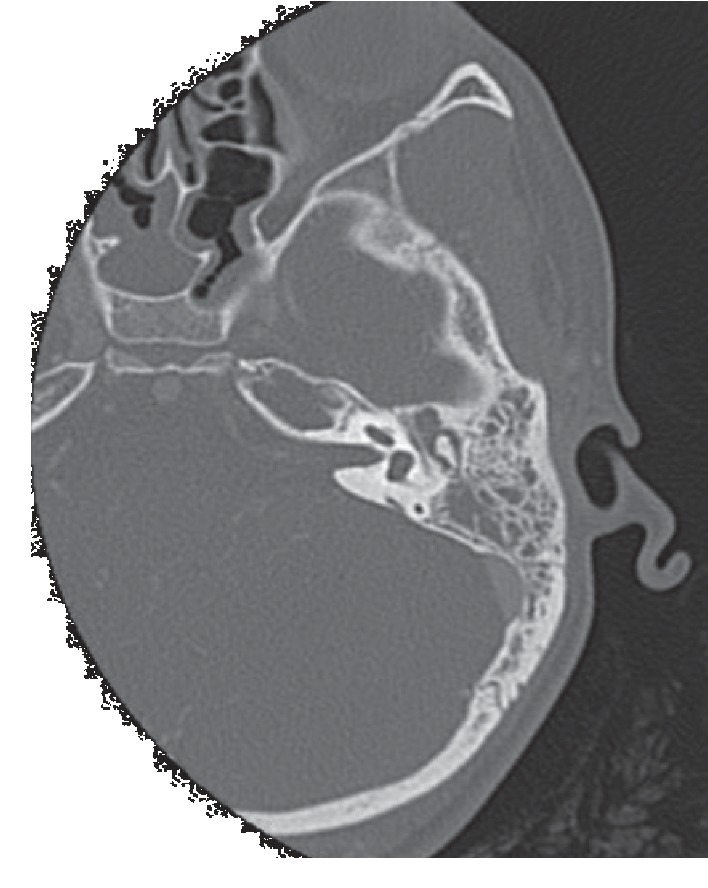
Axial temporal bone computed tomography (CT) scan showing opacification of the left middle ear and mastoid and expanded fluid-filled petrous apex.

The patient underwent contrast-enhanced computed tomography (CT) which revealed left mastoiditis and a left petrous apicitis (apical petrositis) with ipsilateral septic cavernous sinus thrombosis and infectious arteritis and stenosis of the internal carotid artery (Figure 2).

Ophthalmological assessment confirmed left lateral rectus paralysis (Figure 1), with normal biomicroscopy, fundoscopy, and normal visual acuity.

Blood tests revealed elevated inflammatory parameters with a white blood cell (WBC) count of 26200/*μ*L with 24500 neutrophils/*μ*L (93.4%) and C-reactive protein of 88.8 mg/L. Renal function and clotting were unremarkable.

**Figure 3:**
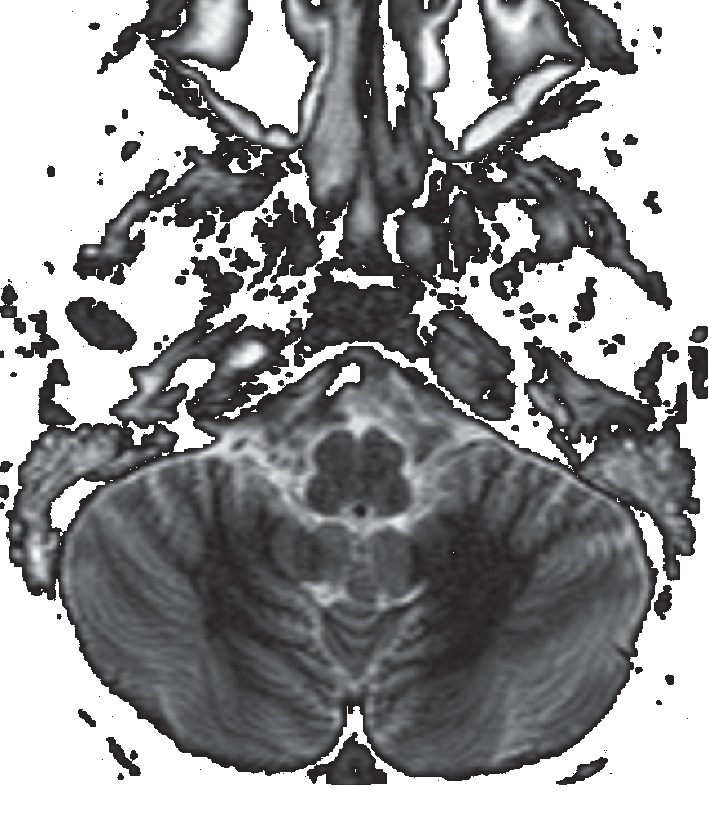
Axial STIR sequence magnetic resonance imaging (MRI) shows a diffuse bilateral inflammatory involvement of middle ear-mastoid with extension of the inflammatory process to petrous apex on the left side.

**Figure 4:**
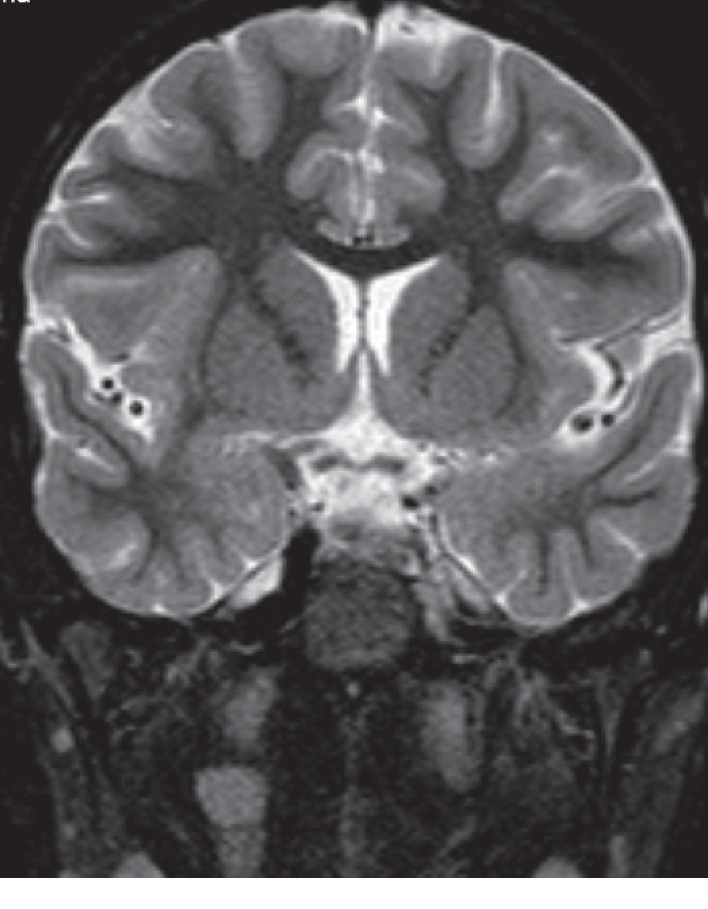
Coronal T2-weighted image shows inflammatory involvement of the left petrous apex and Meckel cavity with petrous carotid stenosis.

**Figure 5:**
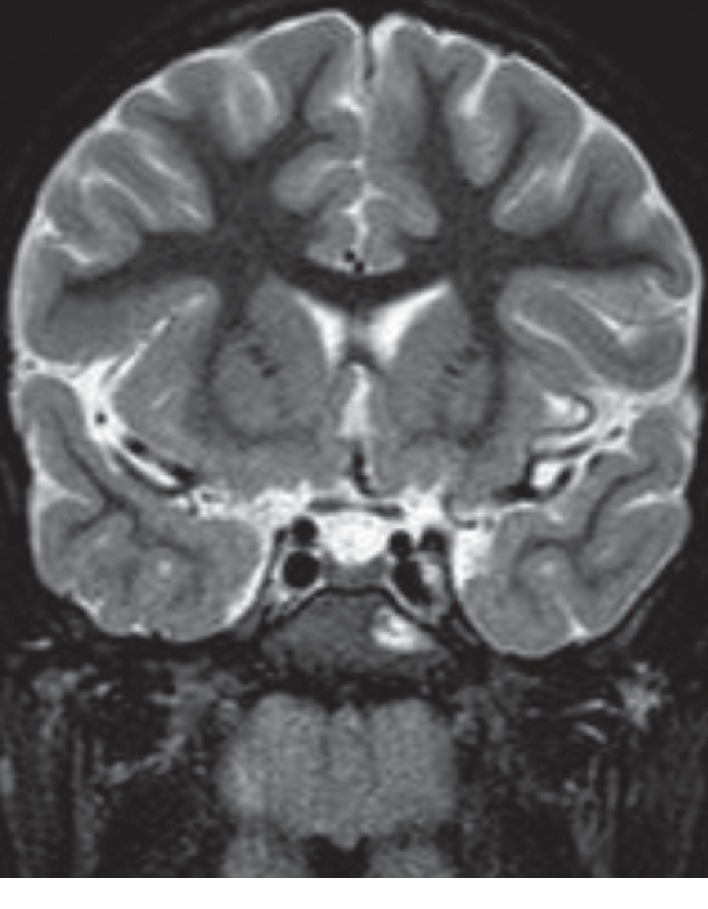
Coronal T2-weighted image reveals an expanded and heterogeneous cavernous sinus.

**Figure 6:**
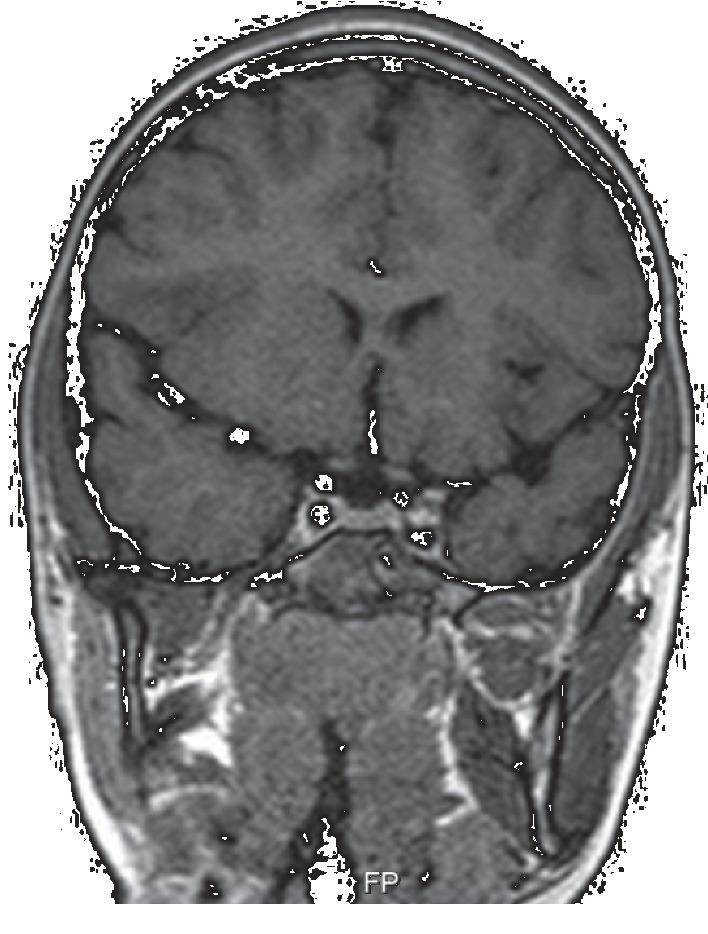
Coronal contrast-enhanced T1-weighted image shows a heterogeneous abnormal enhancement of left cavernous sinus.

**Figure 7:**
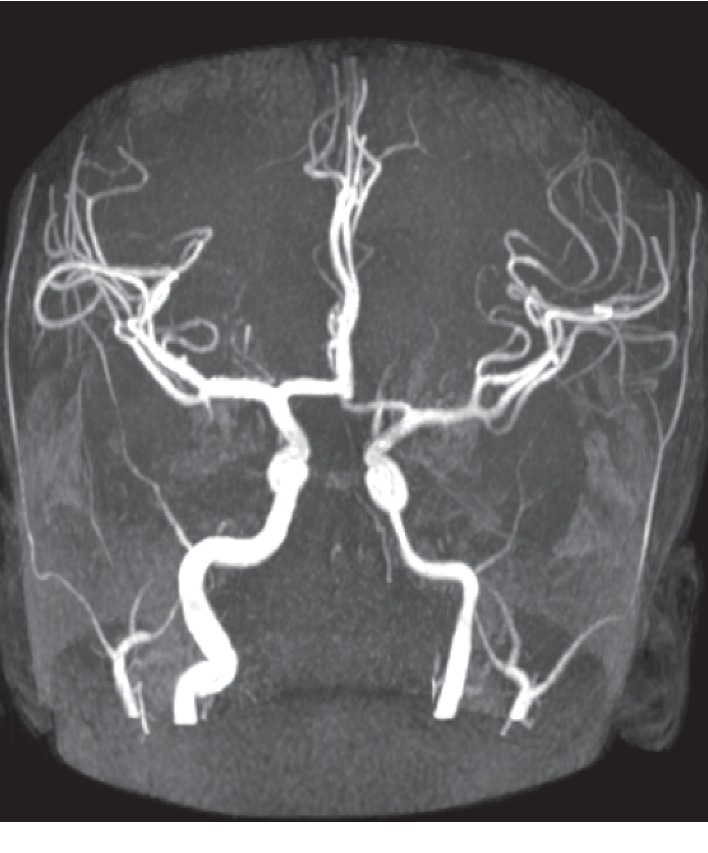
TOF 3D MRI angiography at admission, showing narrowing of the petrous, cavernous, and terminal segments of the left internal carotid artery corresponding to left internal carotid arteritis.

**Figure 8:**
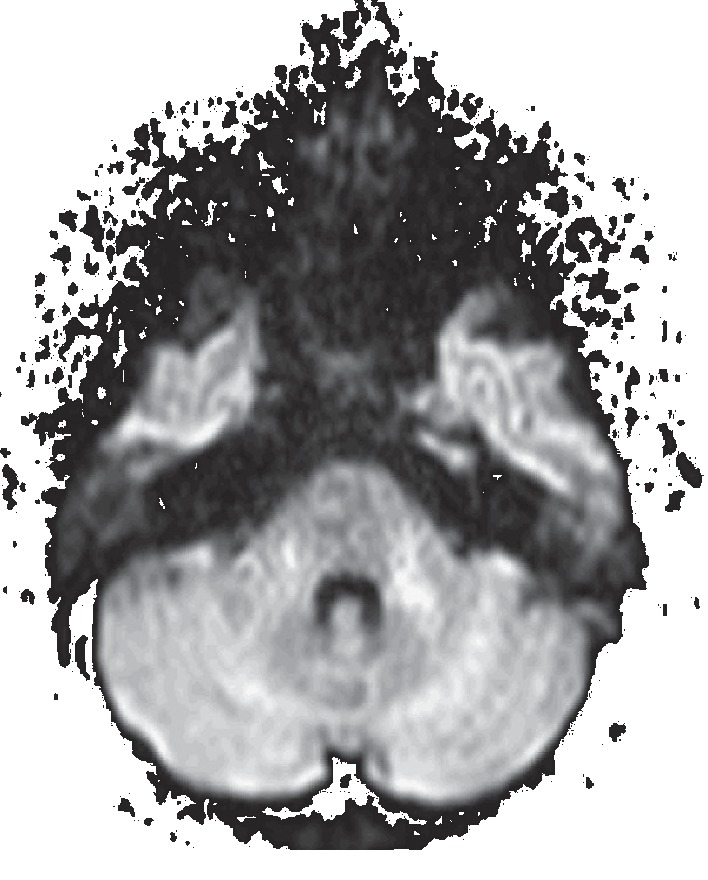
Axial diffusion-weighted imaging revealing the absence of restricted diffusion of water within the cavernous sinus and the temporal bone, excluding the presence of abscess and cholesteatoma.

Magnetic resonance imaging (MRI) showed a diffuse inflammatory enhancement in the left mastoid air cells and the petrous apex with extraosseous extension of the inflammatory process to the petroclival suture and dura adjacent to the clivus and petrous apex on the same side (Figures 3 and 4). Also, inflammatory intense enhancement in the left carotid canal with stenosis of about 50% of the petrous, lacerum, and cavernous segments, and intense enhancement and swelling of the cavernous sinus was seen (Figures 3–8).

The patient was diagnosed with GS based on her clinical presentation, which included acute suppurative otitis media with involvement of the sixth cranial nerve, and based on the evidence of petrous apicitis (apical petrositis) on the CT. The imaging studies also revealed the presence of cavernous sinus thrombosis and carotid septic stenosis. Other differential diagnosis of petrous apicitis including congenital cholesteatoma, intracranial abscess, lateral sinus thrombosis, cholesterol granuloma, temporal bone osteomyelitis, and neoplastic or granulomatous disease were excluded according to the clinical and radiological presentation [10, 11].

Empirical intravenous antibiotic therapy with ceftriaxone (100 mg/kg/day), vancomycin (60 mg/kg/day, with a need to increase the dose to 90 mg/kg/day to reach therapeutic levels), and metronidazole (40 mg/kg/day) was started. Ofloxacin 0.3% ear drops were also initiated. Considering the internal carotid arteritis with significant stenosis, corticoid therapy with methylprednisolone (2 mg/kg/day) and antiplatelet therapy with aspirin (4 mg/kg/day) were decided.

She was submitted to left canal-wall-up mastoidectomy and myringotomy with tube placement with intraoperative findings of abundant purulent middle-ear effusion and granulation tissue in the mastoid cavity and middle ear. Postoperative management included intermediate care unit monitoring for the first week, followed by transfer to the pediatrics ward.

She completed 13 days of ceftriaxone (suspended due to rash) and 21 days of vancomycin and metronidazol with complete resolution of clinical findings. Tapering of methylprednisolone was uneventful. Antiplatelet therapy with aspirin was continued for 3 months.

The abducens nerve palsy totally disappeared 3 days after admission.

The WBC count and C-reactive protein decreased and the microbial culture from the middle-ear fluid isolated *Streptococcus pyogenes* susceptible to penicillin and erythromycin. The blood culture was negative.

**Figure 9:**
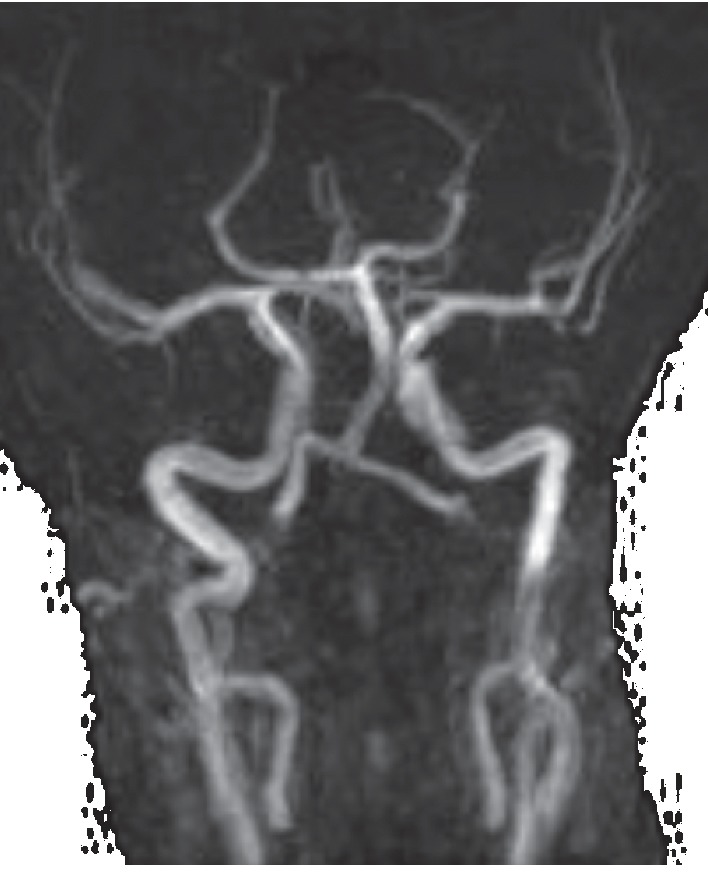
TOF 3D MRI angiography 10 days after treatment reveals total recalibration of the internal carotid artery.

A contrast-enhanced cranial MRI 10 days after admission revealed partial regression of inflammatory signs in the mastoid cells and left petrous apex with total repermeabilization of the carotid artery (Figure 9)

On outpatient follow-up, at 3 months following hospital discharge, she presented complete clinical and radiological remission, without long-term sequelae.

The grommet tube was still in place at consultation, with otherwise normal tympanic membrane. Pure tone audiometry was also normal at 4 months.

## 3. Discussion

We report an atypical and potentially life-threatening case of GS. As previously reported, the typical triad of GS is not always seen, and symptoms should be taken in the context of all the patients' presenting symptoms, signs, and investigations [12].

Our patient did not present facial sensation impairment. Likewise, in Gradenigo's original case series of 57 patients, more than half of the cases did not follow the classical triad [1].

Here, we highlight the importance of recognizing the classical triad and not forgetting GS association with the septic vascular commitment of the cavernous sinus and carotid artery.

Advances in imaging have assisted diagnosis and monitoring of GS [13]. In fact, any child presenting with acute otitis media with suspected intracranial complication should undergo imaging testing. CT scan is the first choice of imaging, since it is widely available and detects abnormalities in bone structures, including destruction of trabecular bone and erosion of the petrous apex. MRI is more sensitive in detecting dural thickening and enhancement as well as intracranial complications and has the advantage of avoiding unnecessary radiation to the patient. A MRI angiography may be performed to rule out signs of sinus thrombosis [4, 13].

The ideal treatment for petrous apicitis is controversial and typically depends on the severity of clinical presentation [14]. Recently, some authors have advocated for nonsurgical intervention with intravenous antibiotic therapy [15]. In our case, surgical treatment was decided, combined with antibiotic therapy due to the severity of clinical presentation with vascular commitment. Indeed, surgical debridement is necessary in very severe cases or cases resistant to medical treatment alone [15]. Another case report by Janjua et al. of a patient with GS with epidural abscess and internal carotid arteritis has described a good response to myringotomy and grommet insertion combined with antibiotic and antiplatelet therapy [10]. However, the authors considered mastoidectomy unnecessary given the partially opacified mastoid air cells in the CT, which was not our case.

Regarding the choice of antibiotics for treatment of GS, most authors advocate for the use of a cephalosporin antibiotic along with metronidazole with or without the addition of vancomycin [10, 11]. Empirical intravenous antibiotics should cover common agents involved in bacterial mastoiditis *(Staphylococcus aureus, Streptococcus pneumoniae, Streptococcus pyogenes, and Pseudomonas aeruginosa*) and anaerobic organisms can also be considered [16, 17]. In our case, ceftriaxone, metronidazol, and vancomycin were chosen preoperatively for empiric broad-spectrum coverage for the most commonly seen organisms. Our case report stands out for its relatively short intravenous antibiotic therapy duration. Previous reports have described variable intravenous antibiotic therapy between 10 and 64 days [12, 15]. Undoubtedly, petrositis caused by infection is equivalent to osteomyelitis, which needs intensive and prolonged antibiotic treatment to avoid relapse [15]. In our case, we considered the 21 days of antibiotic therapy sufficient given the complete resolution of symptoms in the days after surgery.

Considering the MRI finding of internal carotid arteritis, with significant carotid narrowing within the petrous bone, antiplatelet therapy with low-dose aspirin and corticoid therapy with methylprednisolone was decided. However, the decision of using antiplatelet therapy and not using anticoagulant therapy despite the associated finding of intracranial venous thrombosis was not taken lightly. Anticoagulant therapy use is controversial in septic cerebral venous sinus thrombosis in children [18]. A Cochrane review of anticoagulation for cerebral venous sinus thrombosis including two small randomized-controlled trials (79 patients) found a nonsignificant trend towards reduced death and disability in the anticoagulated group [19]. Although there are no pediatric randomized-controlled trials, anticoagulants are often used and seem to be safe and beneficial in cases of cerebral venous sinus thromboses [18–20]. In our patient, given the presence of carotid arteritis, the increased risk of carotid artery rupture and septic embolism with anticoagulation was a concern, having been decided not to initiate hypocoagulation and the use of antiplatelet therapy instead.

## 4. Conclusion

GS is seldom seen in modern medicine, thanks to widespread antibiotic use for the treatment of acute otitis media. However, over the recent decade, the emerging problem of antibiotic resistant bacterial strains should make us alert for potential complications secondary to otitis media [21–23].

Septic vascular commitment of the cavernous sinus and carotid artery is a potentially life-threatening complication of GS. Therefore, prompt recognition and early intervention of petrous apicitis is vital to prevent the consequences of this life-threatening condition. Despite the current trend of conservative management for GS, surgical treatment may still be necessary in severe cases and may shorten the antibiotic therapy duration.

In conclusion, awareness of rare but potentially fatal diagnoses like GS, coupled with prompt investigations is required for early recognition and timely management of our patients.
